# Fibroblast Growth Factor 23 Stimulates Cardiac Fibroblast Activity through Phospholipase C-Mediated Calcium Signaling

**DOI:** 10.3390/ijms23010166

**Published:** 2021-12-23

**Authors:** Ting-Wei Lee, Cheng-Chih Chung, Ting-I Lee, Yung-Kuo Lin, Yu-Hsun Kao, Yi-Jen Chen

**Affiliations:** 1 Division of Endocrinology and Metabolism, Department of Internal Medicine, School of Medicine, College of Medicine, Taipei Medical University, Taipei 11031, Taiwan; b8801138@tmu.edu.tw (T.-W.L.); aglee@tmu.edu.tw (T.-I.L.); 2 Division of Endocrinology and Metabolism, Department of Internal Medicine, Wan Fang Hospital, Taipei Medical University, Taipei 11696, Taiwan; 3 Division of Cardiology, Department of Internal Medicine, School of Medicine, College of Medicine, Taipei Medical University, Taipei 11031, Taiwan; michaelchung110@gmail.com (C.-C.C.); yklin213@yahoo.com.tw (Y.-K.L.); 4 Division of Cardiovascular Medicine, Department of Internal Medicine, Wan Fang Hospital, Taipei Medical University, Taipei 11696, Taiwan; 5 Taipei Heart Institute, Taipei Medical University, Taipei 11031, Taiwan; 6 Department of General Medicine, School of Medicine, College of Medicine, Taipei Medical University, Taipei 11031, Taiwan; 7 Graduate Institute of Clinical Medicine, College of Medicine, Taipei Medical University, Taipei 11031, Taiwan; 8 Department of Medical Education and Research, Wan Fang Hospital, Taipei Medical University, Taipei 11696, Taiwan; 9 Cardiovascular Research Center, Wan Fang Hospital, Taipei Medical University, Taipei 11696, Taiwan

**Keywords:** calcium, fibroblast growth factor 23, fibrosis, inositol 1,4,5-trisphosphate, phospholipase c

## Abstract

Fibroblast growth factor (FGF)-23 induces hypertrophy and calcium (Ca^2+^) dysregulation in cardiomyocytes, leading to cardiac arrhythmia and heart failure. However, knowledge regarding the effects of FGF-23 on cardiac fibrogenesis remains limited. This study investigated whether FGF-23 modulates cardiac fibroblast activity and explored its underlying mechanisms. We performed MTS analysis, 5-ethynyl-2′-deoxyuridine assay, and wound-healing assay in cultured human atrial fibroblasts without and with FGF-23 (1, 5 and 25 ng/mL for 48 h) to analyze cell proliferation and migration. We found that FGF-23 (25 ng/mL, but not 1 or 5 ng/mL) increased proliferative and migratory abilities of human atrial fibroblasts. Compared to control cells, FGF-23 (25 ng/mL)-treated fibroblasts had a significantly higher Ca^2+^ entry and intracellular inositol 1,4,5-trisphosphate (IP_3_) level (assessed by fura-2 ratiometric Ca^2+^ imaging and enzyme-linked immunosorbent assay). Western blot analysis showed that FGF-23 (25 ng/mL)-treated cardiac fibroblasts had higher expression levels of calcium release-activated calcium channel protein 1 (Orai1) and transient receptor potential canonical (TRPC) 1 channel, but similar expression levels of α-smooth muscle actin, collagen type IA1, collagen type Ⅲ, stromal interaction molecule 1, TRPC 3, TRPC6 and phosphorylated-calcium/calmodulin-dependent protein kinase II when compared with control fibroblasts. In the presence of ethylene glycol tetra-acetic acid (a free Ca^2+^ chelator, 1 mM) or U73122 (an inhibitor of phospholipase C, 1 μM), control and FGF-23-treated fibroblasts exhibited similar proliferative and migratory abilities. Moreover, polymerase chain reaction analysis revealed that atrial fibroblasts abundantly expressed FGF receptor 1 but lacked expressions of FGF receptors 2-4. FGF-23 significantly increased the phosphorylation of FGF receptor 1. Treatment with PD166866 (an antagonist of FGF receptor 1, 1 μM) attenuated the effects of FGF-23 on cardiac fibroblast activity. In conclusion, FGF-23 may activate FGF receptor 1 and subsequently phospholipase C/IP_3_ signaling pathway, leading to an upregulation of Orai1 and/or TRPC1-mediated Ca^2+^ entry and thus enhancing human atrial fibroblast activity.

## 1. Introduction

Cardiac fibrosis is a common pathological change in the late stage of most heart disease types and represents a major threat to human health. Substantial evidence indicates that cardiac fibroblasts play a crucial role in the pathogenesis of cardiac fibrosis [1,2]. In response to myocardial injury, quiescent cardiac fibroblasts are activated and can transform into α-smooth muscle actin (α-SMA)-expressing myofibroblasts, participating in the reparative wound healing response through their augmented proliferative, migratory, collagen-producing and contractile abilities. Although activation of myofibroblasts following cardiac injury is essential to preserve the structural integrity of the heart, sustained and excessive myofibroblast activation becomes maladaptive and eventually leads to fibrogenesis [3]. Excessive cardiac fibrosis increases myocardial stiffness and disturbs the mechanical and electrical coupling of cardiomyocytes, thus contributing to a distorted heart architecture, reducing cardiac contraction, and increasing the risk of arrhythmogenesis. These processes eventually lead to the development of overt heart failure and cardiac arrhythmia [1,4].

Fibroblast growth factor (FGF)-23, a member of the FGF family, circulates as an endocrine regulator for phosphate and calcium (Ca^2+^) homeostasis. Moreover, multiple studies have indicated the crucial involvement of FGF-23 in heart failure pathophysiology. A high circulating FGF-23 level was reported to be associated with high risks of cardiovascular diseases, heart failure, and mortality in the elderly population [5]. A clinical investigation suggested that FGF-23 can be a novel risk factor for coronary artery disease and heart failure in the general population [6]. The administration of FGF-23 directly induced left ventricular hypertrophy in mice [7]. FGF-23 activated FGF receptor 4, thus stimulating phospholipase C (PLC)/calcineurin/nuclear factor of the activated T-cell (NFAT) signaling and contribuing to hypertrophic growth of neonatal rat ventricular myocytes [8]. Our previous study indicated that FGF-23 may increase the activity of sarcoplasmic reticulum Ca^2+^-ATPase, a major regulatory protein that extrudes cytosolic Ca^2+^ to the sarcoplasmic reticulum, thereby enhancing the sarcoplasmic reticulum Ca^2+^ content in cardiomyocytes and leading to arrhythmogenesis [9]. However, the effects of FGF-23 on cardiac fibroblasts remain unclear. This study investigated whether FGF-23 can modulate cardiac fibroblast activity and explored the underlying mechanisms.

## 2. Results

### 2.1. Effects of FGF-23 on Cardiac Fibroblast Activity

**Figure 1:**
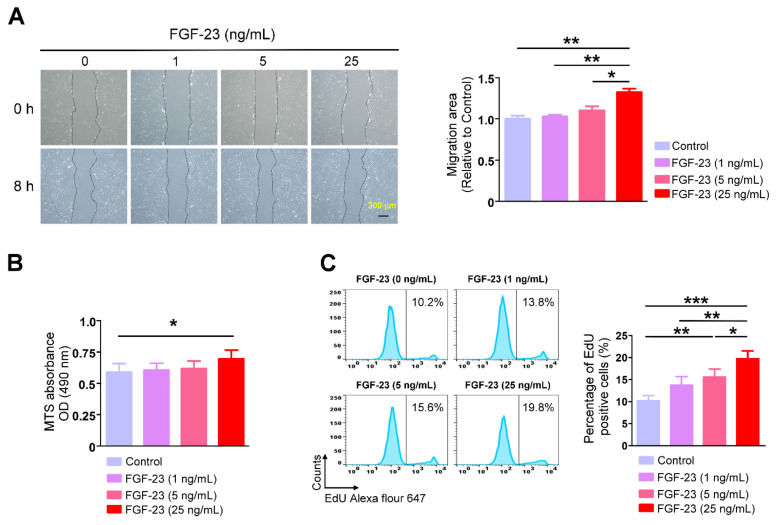
Cardiac fibroblast migration and proliferation in control and fibroblast growth factor (FGF)-23-treated human atrial fibroblasts. (**A**) Representative images (left panel) and average data (right panel, *n* = 5) of the migration assay of cardiac fibroblasts. Confluent cells were scratched 8 h before the endpoint of treatments, and images were taken at 0 h and 8 h post wound from control and FGF-23 (1, 5 and 25 ng/mL)-treated cardiac fibroblasts. Scale bar represents 300 μm. (**B**) Average data of cell proliferation analysis with the MTS reagent (*n* = 5). The optical density (OD) value was measured at a wavelength of 490 nm in cardiac fibroblasts treated without and with FGF-23 (1, 5 and 25 ng/mL) for 48 h. (**C**) Representative images (left panel), average data and statistical differences (right panel, *n* = 5) of an 5-ethynyl-2′-deoxyuridine (EdU)-based flow cytometry assay to evaluate cell proliferation in control and FGF-23 (1, 5 and 25 ng/mL)-treated cardiac fibroblasts. * *p* < 0.05, ** *p* < 0.01, *** *p* < 0.005.

**Figure 2:**
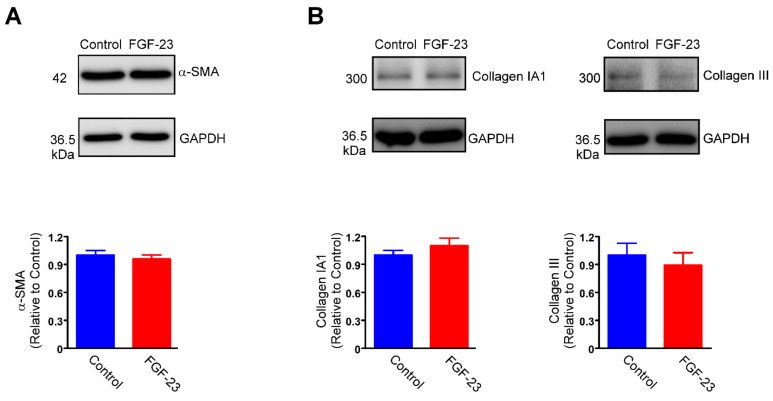
The α-smooth muscle actin (α-SMA) expression and collagen production in control and fibroblast growth factor (FGF)-23-treated human atrial fibroblasts. Representative immunoblots and average data of α-SMA (**A**), collagen type IA1 and collagen type Ⅲ (**B**) from control and FGF-23 (25 ng/mL)-treated cardiac fibroblasts (*n* = 5).

We evaluated the effects of FGF-23 (1, 5 and 25 ng/mL) on human atrial fibroblasts and found that FGF-23 (25 ng/mL, but not 1 or 5 ng/mL)-treated cardiac fibroblasts had a significantly higher migratory ability than did control cells (Figure 1A). MTS assay demonstrated that FGF-23 (25 ng/mL)-treated cardiac fibroblasts had a considerably higher optical density (OD) value than did control cells (Figure 1B). The succinate dehydrogenase activity measured by MTS assay indicates differences in cell proliferation, viability, or enzyme function. 5-ethynyl-2′-deoxyuridine (EdU) is a nucleoside analog to thymidine and is incorporated into DNA during active DNA synthesis. Similarly, EdU-based proliferation assay revealed that FGF-23 (25 ng/mL)-treated cardiac fibroblasts had a greater number of positive EdU staining cardiac fibroblasts than did control cells (Figure 1C). These findings suggest that the effects of FGF-23 on fibroblasts detected by MTS may majorly arise from FGF-23-induced cell proliferation. However, control and FGF-23 (1 ng/mL)-treated cardiac fibroblasts had a similar OD value measured by MTS assay and a similar percentage of positive staining cells assessed by EdU assay. Additionally, treatment with FGF-23 at 5 ng/mL significantly increased the percentage of EdU-positive cells compared to control fibroblasts. FGF-23 (25 ng/mL)-treated cardiac fibroblasts had a higher percentage of EdU-positive cells than did FGF-23 (5 ng/mL)-treated cardiac fibroblasts. These findings imply that FGF-23 concentration-dependently upregulates cardiac fibroblasts activity. Differently, there was no significant difference observed in protein expression level of α-SMA between control and FGF-23 (25 ng/mL)-treated cardiac fibroblasts (Figure 2A). Control and FGF-23 (25 ng/mL)-treated cardiac fibroblasts had similar protein expressions of collagen type IA1 and collagen type Ⅲ (Figure 2B).

### 2.2. FGF-23 Promotes Cardiac Fibroblast Activity through FGF Receptor 1 Activation

**Figure 3:**
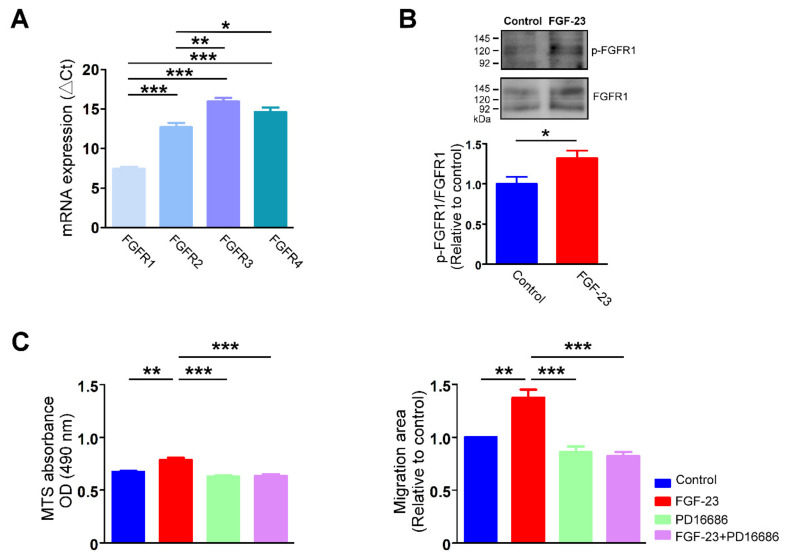
Fibroblast growth factor (FGF)-23 stimulates cardiac fibroblast proliferation and migration through the activation of FGF receptor 1. (**A**) FGF receptor 1–4 mRNA expressions in human atrial fibroblasts (*n* = 5) using RT-qPCR. Relative mRNA abundance of FGF receptor 1–4 was estimated from the threshold cycle (Ct) value and normalized to the respective Ct value of glyceraldehyde 3-phosphate dehydrogenase (∆Ct). (**B**) FGF receptor 1 phosphorylation analysis using immunoblotting from cells stimulated with FGF-23 (25 ng/mL) for 15 min (*n* = 5). (**C**) Average data of a MTS assay to study cell proliferation (left panel) and a wound-healing assay to evaluate cell migration (right panel) in control and FGF-23 (25 ng/mL)-treated cardiac fibroblasts without and with PD166866 (a FGF receptor 1 antagonist, 1 μM) treatment (*n* = 5). * *p* < 0.05, ** *p* < 0.01, *** *p* < 0.005.

The expression profile of four distinct FGF receptors (1–4) in cardiac fibroblasts was determined through quantitative polymerase chain reaction (PCR). The results revealed that human atrial fibroblasts abundantly expressed FGF receptor 1 but lacked the expressions of FGF receptors 2–4 (Figure 3A). FGF-23 (25 ng/mL)-treated cardiac fibroblasts demonstrated significantly increased ratios of phosphorylated (p)-FGF receptor 1 to FGF receptor 1 levels compared with control cells (Figure 3B). Moreover, MTS assay showed that PD166866 (1 μM), an FGF receptor 1 antagonist, significantly diminished the increased OD values of FGF-23 (25 ng/mL)-treated cardiac fibroblasts (Figure 3C). Control cells, fibroblasts treated with PD166866 (1 μM) and fibroblasts treated with FGF-23 (25 ng/mL) combined with PD166866 (1 μM) had a similar migratory ability (Figure 3C). These findings suggest that the inhibition of FGF receptor 1 abolished the effects of FGF-23 on cardiac fibroblast activity.

### 2.3. Ca^2+^ Underlying the Effects of FGF-23 on Cardiac Fibroblast Activity

**Figure 4:**
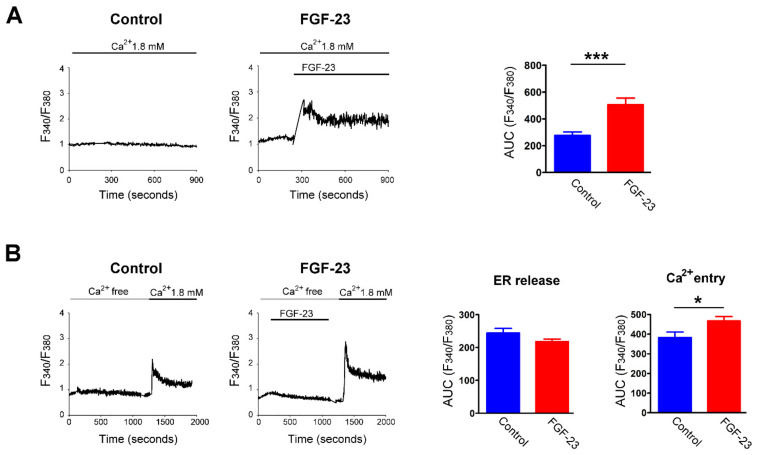
Fibroblast growth factor (FGF)-23 induces a rapid increase in intracellular calcium (Ca^2+^) concentration through enhancing Ca^2+^ entry. (**A**) Representative fura-2 fluorescence tracings (left panel) indicates intracellular Ca^2+^ transient of an individual cardiac fibroblast in the presence of extracellular Ca^2+^ (1.8 mM) without and with FGF-23 (25 ng/mL) treatment. Right panel shows the changes of intracellular Ca^2+^ concentrations in cardiac fibroblast (*n* = 12 cells) quantified as the area under the curve (AUC) values of fluorescence intensities (F340/F380). (**B**) Representative fura-2 fluorescence tracings (left panel) and changes of intracellular Ca^2+^ concentrations (right panel, *n* = 12 cells) in control and FGF-23-treated cardiac fibroblasts. Cells were initially incubated in Ca^2+^-free buffer, and then FGF-23 (25 ng/mL) were added in the absence of extracellular Ca^2+^ to measure endoplasmic reticulum (ER) Ca^2+^ release. Subsequently, FGF-23 was removed from the bath and extracellular Ca^2+^ concentration was restored to 1.8 mM to evaluate extracellular Ca^2+^ entry. The Ca^2+^ changes were quantified as the AUC values of F340/F380 corresponding to ER Ca^2+^ release and extracellular Ca^2+^ entry. * *p* < 0.05, *** *p* < 0.005.

**Figure 5:**
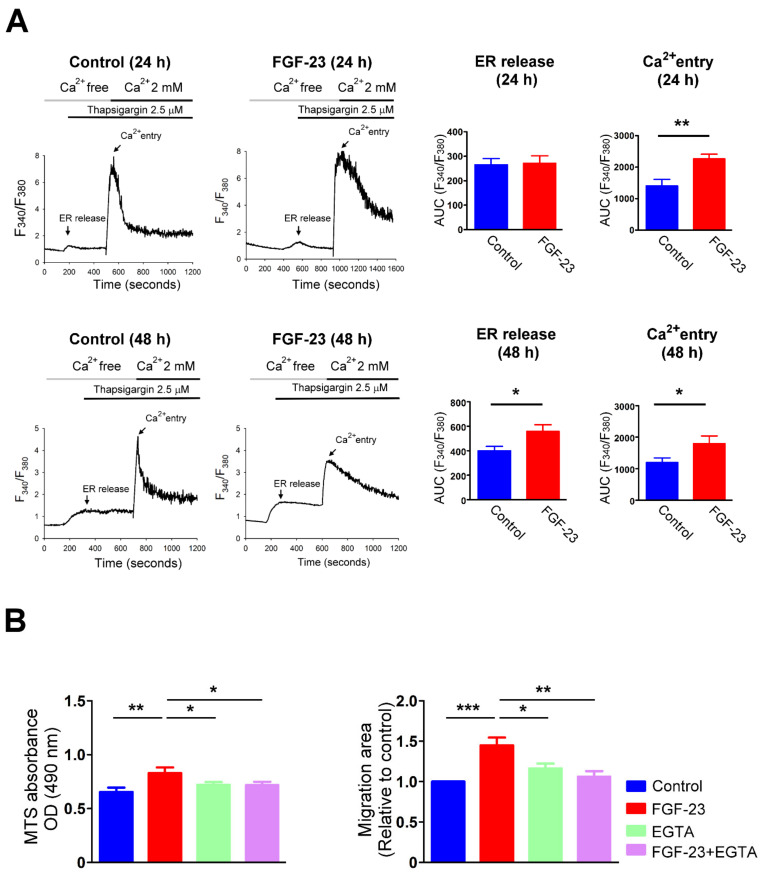
Intracellular calcium (Ca^2+^) mediates the stimulatory effects of fibroblast growth factor (FGF)-23 on cardiac fibroblast proliferation and migration. (**A**) Representative fura-2 fluorescence tracings (left panel) indicates intracellular Ca^2+^ transient after addition of 2.5 μM thapsigargin (an endoplasmic reticulum (ER) Ca^2+^-ATPase inhibitor), and 2 mM Ca^2+^ to an individual cardiac fibroblast without and with FGF-23 (25 ng/mL) treatment for 24 h and 48 h. Thus, the increases of Ca^2+^ levels in cardiac fibroblasts following exposure to FGF-23 are not due to there being more cells present. The average Ca^2+^ changes (right panel, *n* = 12 cells) were quantified as the area under the curve (AUC) values of fluorescence intensities (F340/F380) corresponding to ER Ca^2+^ release and extracellular Ca^2+^ entry. (**B**) Average data of a MTS assay to study cell proliferation (left panel) and a wound-healing assay to evaluate cell migration (right panel) in control and FGF-23 (25 ng/mL)-treated cardiac fibroblasts without and with ethylene glycol tetra-acetic acid (EGTA, a free Ca^2+^ chelator, 1 mM) treatment (*n* = 5). * *p* < 0.05, ** *p* < 0.01, *** *p* < 0.005.

Treatment with FGF-23 (25 ng/mL) rapidly elevated intracellular Ca^2+^ levels in the presence of extracellular Ca^2+^ at 1.8 mM (Figure 4A), but did not elicit an increase in intracellular Ca^2+^ levels without extracellular Ca^2+^ in human atrial fibroblasts (Figure 4B). Moreover, restoration of extracellular Ca^2+^ concentration to 1.8 mM caused a significantly higher intracellular Ca^2+^ levels in FGF-23 (25 ng/mL)-treated human atrial fibroblasts than did control cells (Figure 4B). In a Ca^2+^-free condition, control and FGF-23 (25 ng/mL)-treated cardiac fibroblasts were found to have a similar intracellular Ca^2+^ content at baseline. Following treatment with thapsigargin, an endoplasmic reticulum (ER) Ca^2+^-ATPase inhibitor, control and FGF-23 (25 ng/mL for 24 h)-treated cardiac fibroblasts had a similar ER Ca^2+^ release (Figure 5A). FGF-23 (25 ng/mL for 24 h)-treated cardiac fibroblasts had a significantly higher Ca^2+^ entry after shifting from Ca^2+^-free to 2 mM Ca^2+^ superfusate when compared with control cells. However, FGF-23 (25 ng/mL for 48 h)-treated cardiac fibroblasts had both significantly higher ER Ca^2+^ release and Ca^2+^ entry than did control cells. Moreover, FGF-23 (25 ng/mL for 24 h)-treated cardiac fibroblasts had a significantly higher steady-state of intracellular Ca^2+^ level than control cells (F340/F380 ratio 2.9 ± 0.1 versus 1.9 ± 0.1, *p* < 0.001). However, FGF-23 (25 ng/mL for 48 h)-treated cardiac fibroblasts had an insignificant higher steady-state of intracellular Ca^2+^ level than control cells (F340/F380 ratio 2.3 ± 0.3 versus 1.8 ± 0.1, *p* = 0.10). These findings suggest that FGF-23 augments Ca^2+^ entry, leading to the elevation of intracellular Ca^2+^ level, which stimulates downstream signaling and consequent fibroblast activation. Additionally, FGF-23 may enhance Ca^2+^ reuptake into ER to normalize the intracellular Ca^2+^ levels. We also found that ethylene glycol tetra-acetic acid (EGTA), a free Ca^2+^ chelator, significantly reduced the stimulatory effects of FGF-23 on cardiac fibroblast activity. Control cells, cardiac fibroblasts treated with EGTA (1 mM) and FGF-23 (25 ng/mL) combined with EGTA (1 mM)-treated cardiac fibroblasts had a similar OD value measured by MTS assay (Figure 5B). Likewise, the cell migratory ability was similar among control, EGTA (1 mM)-treated and FGF-23 (25 ng/mL) combined with EGTA (1 mM)-treated cardiac fibroblasts (Figure 5B).

### 2.4. FGF-23 Increases Store-Operated Ca^2+^ Entry in Cardiac Fibroblasts

**Figure 6:**
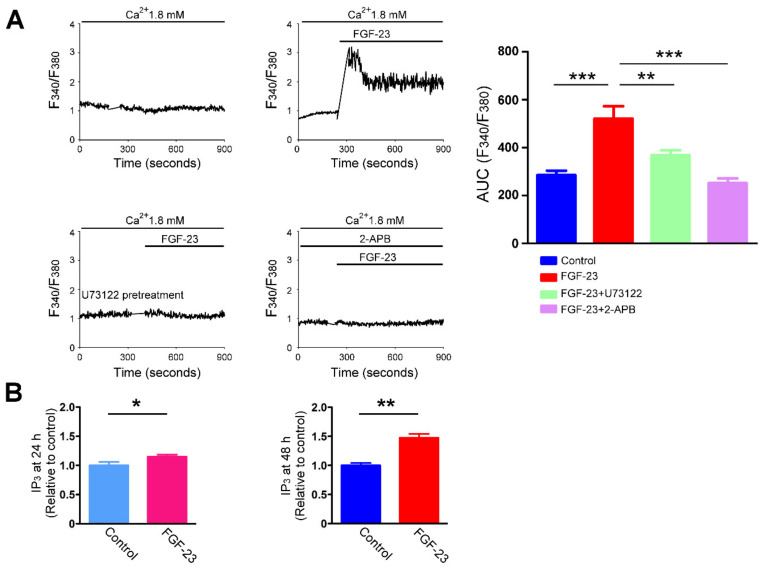
Fibroblast growth factor (FGF)-23 stimulates inositol 1,4,5-trisphosphate (IP_3_) production in cardiac fibroblasts. (**A**) Representative fura-2 fluorescence tracings (left panel) indicates intracellular Ca^2+^ transient of an individual cardiac fibroblast in the presence of extracellular Ca^2+^ (1.8 mM) without and with pretreatment of U73122 (a phospholipase C inhibitor, 1 μM for 5 min) or 2-APB (an IP_3_ inhibitor, 50 uM added 5 min prior to recording), followed by the addition of FGF-23 (25 ng/mL). The changes of intracellular Ca^2+^ concentrations (right panel) in cardiac fibroblasts (*n* = 12 cells) were quantified as the area under the curve (AUC) values of fluorescence intensities (F340/F380). (**B**) Average data of intracellular IP_3_ levels in cardiac fibroblasts without and with FGF-23 (25 ng/mL) treatment for 24 h (left panel, *n* = 5) and 48 h (right panel, *n* = 5). Data were expressed as a fold change relative to control. * *p* < 0.05, ** *p* < 0.01, *** *p* < 0.005.

**Figure 7:**
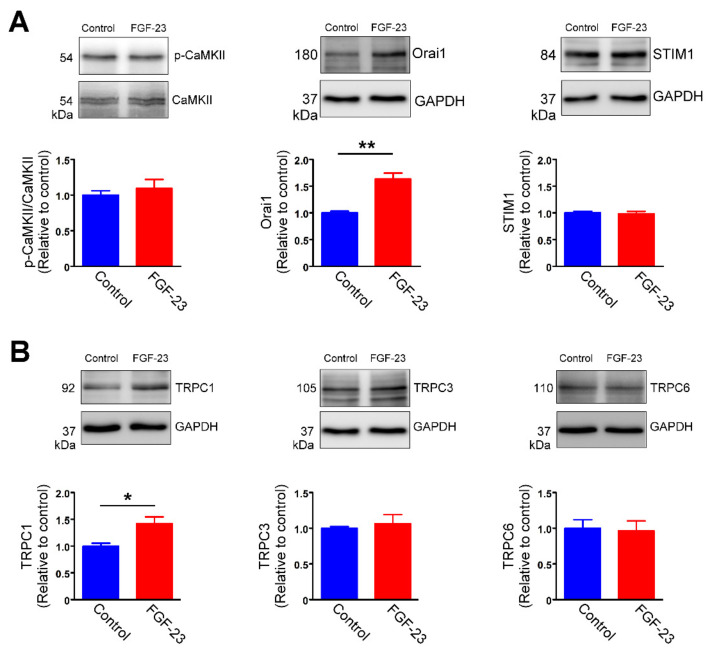
Fibroblast growth factor (FGF)-23 increases store-operated calcium channel expression in cardiac fibroblasts. Representative immunoblots and average data of phosphorylated-calcium/calmodulin-dependent protein kinase II (p-CaMKⅡ), calcium release-activated calcium channel protein 1 (Orai1), stromal interaction molecule 1 (STIM1) (**A**), and transient receptor potential canonical (TRPC) 1 channel, TRPC3 and TRPC6 (**B**) expression in control and FGF-23 (25 ng/mL)-treated cardiac fibroblasts (*n* = 5). * *p* < 0.05, ** *p* < 0.01.

To assess whether FGF-23 results in rapid inositol 1,4,5-trisphosphate (IP_3_) production, we measured FGF-23-induced changes in intracellular Ca^2+^ levels in the absence and presence of U73122 (a PLC inhibitor) and 2-aminoethoxydiphenyl borate (2-APB, an IP_3_ inhibitor). The rapid increase in intracellular Ca^2+^ levels induced by FGF-23 was abolished by pretreatment of U73122 (1 μM) and 2-APB (50 uM), respectively (Figure 6A). As shown in Figure 6B, treatment with FGF-23 (25 ng/mL) for 24 h and 48 h both obviously increased intracellular inositol 1,4,5-trisphosphate (IP_3_) levels in cardiac fibroblasts. However, control and FGF-23 (25 ng/mL)-treated cardiac fibroblasts had similar phosphorylation levels of calcium/calmodulin-dependent protein kinase II (CaMKⅡ), suggesting that FGF-23 did not activate CaMKⅡ signaling in cardiac fibroblasts (Figure 7A). Since FGF-23 increased IP_3_ levels and Ca^2+^ entry, we studied the effects of FGF-23 on store-operated Ca^2+^ channels. FGF-23 (25 ng/mL)-treated cardiac fibroblasts had higher protein expression levels of calcium release-activated calcium channel protein 1 (Orai1), but had similar protein expression levels of stromal interaction molecule 1 (STIM1) when compared with control cells (Figure 7A). Additionally, FGF-23 (25 ng/mL)-treated cardiac fibroblasts had increased protein expression levels of transient receptor potential canonical (TRPC) 1 channel than did control fibroblasts (Figure 7B). The protein expression levels of TRPC3 and TRPC6 were similar between FGF-23 (25 ng/mL)-treated cardiac fibroblasts and control cells.

### 2.5. PLC Signaling Mediates the Effects of FGF-23 on Cardiac Fibroblast Activity

**Figure 8:**
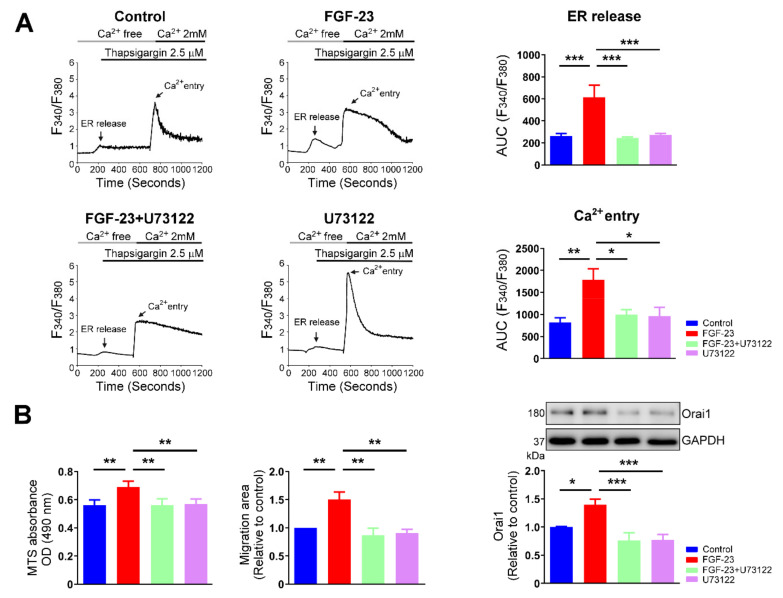
Phospholipase C (PLC) signaling involved in fibroblast growth factor (FGF)-23 stimulates cardiac fibroblast proliferation and migration. (**A**) Representative fura-2 based intracellular calcium (Ca^2+^) tracings (left panel) and average changes in Ca^2+^ concentrations (right panel, *n* = 12 cells) from control, FGF-23 (25 ng/mL for 48 h)-treated, U73122 (a PLC inhibitor, 1 μM for 48 h)-treated and FGF-23 combined with U73122-treated cardiac fibroblasts. Cells were initially incubated in Ca^2+^-free buffer. The ER Ca^2+^ release and extracellular Ca^2+^ entry were induced after addition of 2.5 μM thapsigargin and subsequently 2 mM Ca^2+^ to the calcium free bath solution, respectively. The average Ca^2+^ changes were quantified as the area under the curve (AUC) values of fluorescence intensities (F340/F380) corresponding to ER Ca^2+^ release and extracellular Ca^2+^ entry. Fura-2 fluorescent signals were recorded from an individual cardiac fibroblast. Thus, the increases of Ca^2+^ levels in cardiac fibroblasts following exposure to FGF-23 should not be caused by there being more cells present. (**B**) Average data of cardiac fibroblast proliferation (left panel, *n* = 5), migration (middle panel, *n* = 5), and representative immunoblots and average data of calcium release-activated calcium channel protein 1 (Orai1) expression (right panel, *n* = 6) from control and FGF-23 (25 ng/mL for 48 h)-treated cardiac fibroblasts without and with U73122 (1 μM) treatment. * *p* < 0.05, ** *p* < 0.01, *** *p* < 0.005.

Treatment with U73122 (1 μM) significantly mitigated the highly elevated ER Ca^2+^ release induced by FGF-23 stimulation in cardiac fibroblasts (Figure 8A). Ca^2+^ entry was similar among control, U73122 (1 μM)-treated and FGF-23 (25 ng/mL) combined with U73122 (1 μM)-treated cardiac fibroblasts. Likewise, control, U73122 (1 μM)-treated fibroblasts, and FGF-23 (25 ng/mL) combined with U73122 (1 μM)-treated fibroblasts had similar Orai1 expressions (Figure 8B). In addition, MTS assay revealed that control cells, fibroblasts treated with U73122 (1 μM) and fibroblasts treated with FGF-23 (25 ng/mL) combined with U73122 (1 μM) had a similar OD value (Figure 8B). We also found that the stimulatory effect of FGF-23 on cardiac fibroblast migration was attenuated by U73122 (Figure 8B). Overall, these findings demonstrate that PLC signaling mediates the effects of FGF-23 on cardiac fibroblast activity.

## 3. Discussion

**Figure 9:**
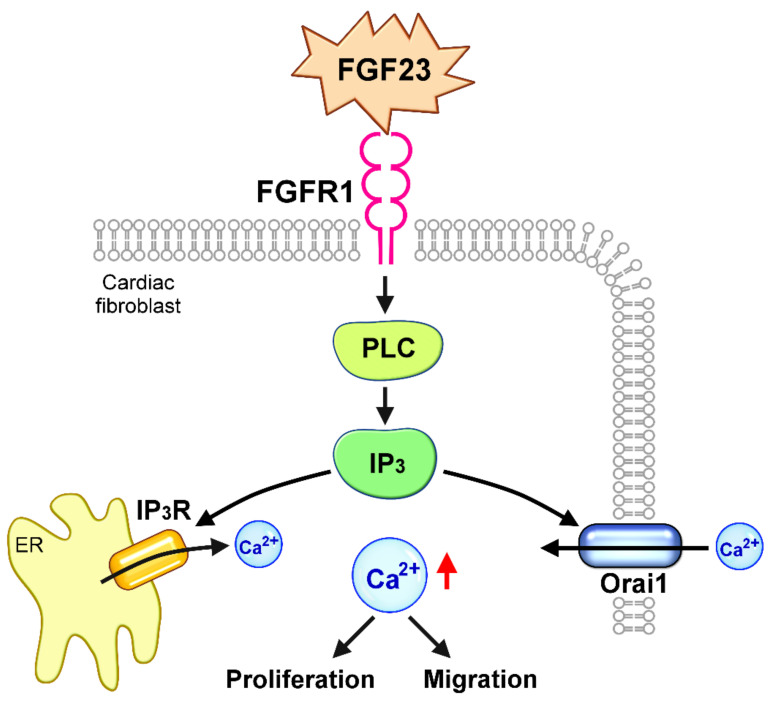
Schematic illustration of the proposed mechanisms underlying the effects of fibroblast growth factor (FGF)-23 on cardiac fibroblast activity. FGF-23 activates FGF receptor 1 (FGFR1) and subsequent phospholipase C (PLC)/ inositol 1,4,5-trisphosphate (IP_3_) signaling pathway, leading to an upregulation of calcium release-activated calcium channel protein 1 (Orai1)-mediated calcium (Ca^2+^) entry and IP_3_ receptor (IP_3_R)-mediated Ca^2+^ release from endoplasmic reticulum (ER), thus stimulating cardiac fibroblast proliferation and migration.

Although FGF-23 has been shown to stimulate the proliferation and migration of neonatal rat cardiac fibroblasts [10,11], mechanisms underlying FGF-23-induced fibrogenetic effects remain unclear. For the first time, we found that FGF-23 significantly promoted cell proliferation and migration through the activation of FGF receptor 1 in human atrial fibroblasts. As shown in the illustration (Figure 9), this study demonstrated that FGF-23 may activate the PLC/IP_3_ signaling pathway, leading to an upregulation of Orai1-mediated Ca^2+^ entry and thus enhancing cardiac fibroblast activity. Accordingly, FGF-23 may be a potential target for regulating cardiac fibrosis, especially in patients with chronic kidney disease who generally have higher circulating FGF-23 levels.

A previous study has shown that the mean plasma concentrations of FGF-23 were 20 ± 5 ng/mL in peritoneal dialysis patients [12]. Treatment with FGF-23 at 100 ng/mL significantly enhanced proliferation and migration of neonatal rat cardiac fibroblasts [10]. Another study reported that FGF-23 dose-dependently increased proliferation of adult mouse cardiac fibroblasts. Both 25 and 50 ng/mL FGF-23 stimulated fibroblast proliferation to a similar extent [13]. Hence, we evaluated the effect of FGF-23 at clinically relevant concentrations (1, 5 and 25 ng/mL), and found that FGF-23 only increased atrial fibroblast activity at 25 ng/mL. This study used recombinant mouse FGF-23 to stimulate human cardiac fibroblasts, though mouse FGF-23 has been reported to bear 72% amino acid homology to human FGF-23 [14]. Human cardiac fibroblasts might be supposed to produce a greater response to human FGF-23. Additionally, our experiments demonstrated that FGF-23 (25 ng/mL)-treated cardiac fibroblasts exhibited a significant increase (around 32%) in migration when compared with control fibroblasts. However, MTS and EdU incorporation assays showed that FGF-23 (25 ng/mL) only enhanced cardiac fibroblast proliferation with the extents of 17% and 19% as compared to control cells. These findings suggest that the differences in wound closure might arise from the effect of FGF-23 on fibroblast migration in addition to its effect on cell proliferation.

Laboratory evidence indicated that FGF-23 directly induces left ventricular hypertrophy through the activation of the FGF receptor [7]. The mammalian genome encodes four FGF receptor isoforms, FGF receptors 1–4 [15]. Expressions of different FGF receptor isoforms are tissue specific. The immunohistochemical analysis of normal human tissues illustrated the predominant expression of FGF receptor 1 and FGF receptor 4 in cardiomyocytes [16]. Although the expression levels were lower when compared with FGF receptor 1, multiple studies have indicated that FGF receptor 4 mediated the pro-hypertrophic effect of FGF-23 on the heart [8,17]. However, the FGF receptor isoform expression pattern in cardiac fibroblasts remains unknown. A flow cytometry analysis of the adult mouse heart revealed that FGF receptor 4 is mainly expressed in cardiac myocytes, not in fibroblasts [8]. Our PCR analysis results demonstrated that in human atrial fibroblasts, FGF receptor 1 was markedly expressed, with barely detectable expressions of FGF receptors 2–4. Thus, FGF receptors 2–4 may play limited roles in mediating the effects of FGF-23 on human atrial fibroblasts. However, western blot analysis of all FGF receptors would help to convince that FGF receptor 1 and not FGF receptors 2–4 is expressed in human atrial fibroblasts in our experiments. Furthermore, our findings revealed that treatment with FGF-23 significantly increased FGF receptor 1 phosphorylation in cardiac fibroblasts. We also found that the inhibition of FGF receptor 1 with PD166866 significantly attenuated the enhanced proliferatory and migratory abilities of FGF-23-treated fibroblasts. These results suggest that FGF-23 increases atrial fibroblast activity through the activation of FGF receptor 1. FGFR1 is also known to signal through mitogen-activated protein kinase and protein kinase B (Akt) [18]. We found that the phosphorylation levels of extracellular signal-regulated kinase (ERK) and Akt were similar between FGF-23 (25 ng/mL for 15 min)-treated cardiac fibroblasts and control cells (Supplementary Figure S1). These findings suggest that the effects of FGF-23 on human atrial fibroblast activity are not associated with the action of ERK or Akt signaling.

Exposure of primary mouse cardiomyocytes to FGF-23 led to an elevated intracellular Ca^2+^ level, which has been linked to a high risk of cardiac hypertrophy [19]. FGF-23 may promote arrhythmogenesis through increasing the Ca^2+^ content and Ca^2+^ leak in cardiomyocytes [9,20]. Moreover, intracellular Ca^2+^ is a critical regulator of multiple functions of cardiac fibroblasts [21]. Our previous studies have demonstrated that the upregulation of the intracellular Ca^2+^ level contributed to increased atrial fibroblast activity [22,23,24]. In this study, we demonstrated that treatment with FGF-23 stimulated IP_3_ production and increased Ca^2+^ entry in human atrial fibroblasts. In response to augmented Ca^2+^ entry, ER Ca^2+^-ATPase would be activated to operatively suppress the increasing cytosolic Ca^2+^ levels. We found that FGF-23 increased protein expression levels of ER Ca^2+^-ATPase in cardiac fibroblasts (Supplementary Figure S2), which may reduce the cytosolic Ca^2+^ levels after increasing Ca^2+^ entry by FGF-23. In addition, FGF-23 (25 ng/mL for 48 h)-treated cardiac fibroblasts also had considerably higher ER Ca^2+^ stores than FGF-23 (25 ng/mL for 24 h)-treated and control fibroblasts. However, an increased level of ER Ca^2+^-ATPase protein expression may not necessarily indicate enhanced reaction activity. Measurements of ER Ca^2+^-ATPase reaction rate could provide the direct evidence to support this conclusion. Moreover, the upregulated cell proliferative and migratory abilities of FGF-23-treated cardiac fibroblasts were eliminated by EGTA. Our results imply that FGF-23 modulated Ca^2+^ homeostasis to promote human atrial fibroblast activity.

Our previous investigations have reported that FGF-23 increased arrhythmogenesis through the activation of CaMKII signaling in atrial myocytes and pulmonary vein cardiomyocytes [9,20]. In this study, we found that FGF-23 did not affect the phosphorylation of CaMKII, suggesting that CaMKII is not involved in the effects of FGF-23 on proliferative and migratory abilities in human atrial fibroblasts. FGF-23 has been demonstrated to phosphorylate PLC, which activates Ca^2+^/calcineurin/NFAT pathway, ultimately leading to cardiomyocyte hypertrophy [8]. Moreover, calcineurin is involved in the signal transduction of angiotensin II and norepinephrine-induced cardiac fibroblast activation [25,26]. However, it remains to be explored whether calcineurin/NFAT signaling may play a role in the stimulatory effects of FGF-23 on cardiac fibroblasts. Additionally, the major Ca^2+^ entry pathway in cardiac fibroblasts is store-operated Ca^2+^ entry [21,27]. PLC activation hydrolyzes phosphatidylinositol 4,5-bisphosphate to generate diacylglycerol and IP_3_. IP_3_ induces Ca^2+^ release from ER via the IP_3_ receptor. The depletion of Ca^2+^ stores in ER leads to the activation of STIM1 which is the Ca^2+^ sensing protein distributed mainly on the surface of the ER. Activated STIM1 will then translocate to the plasma membrane where it can interact with Orai1, the plasma membrane Ca^2+^ channel, inducing store-operated Ca^2+^ entry [21,28]. Ventricular fibroblasts derived from patients with heart failure exhibited increased Orai1, but similar STIM1 protein expression levels compared to those of non-failing hearts [29]. Inhibition of Orai1 ameliorated left ventricular systolic dysfunction in mice subjected to transverse aortic constriction-induced pressure overload. The cardioprotective effects of Orai1 inhibition were associated with a reduction in cardiac fibrosis and prevention of Ca^2+^ homeostasis alterations [30]. In the present study, the acute Ca^2+^ response to FGF-23 in human atrial fibroblasts showed that intracellular Ca^2+^ level was not elevated in the absence of extracellular Ca^2+^, suggesting that FGF-23 induces a rapid increase in intracellular Ca^2+^ levels through stimulating Ca^2+^ entry. In addition, 2-APB is a chemical that blocks store-operated Ca^2+^ entry and IP_3_-induced Ca^2+^ release [31]. However, 2-APB at 50 μM (the concentration used in this study), has been shown to act as an IP_3_ inhibitor [32]. Treatment with a PLC inhibitor or an IP_3_ inhibitor abolished the FGF-23-evoked increases in the intracellular Ca^2+^ levels, implying that FGF-23 actually results in IP_3_ production. Moreover, the FGF-23-induced upregulations of Orai1 expression and cardiac fibroblast activity were both suppressed by PLC inhibition. Accordingly, our findings revealed that FGF-23 enhances store-operated Ca^2+^ entry in cardiac fibroblasts through activating PLC/IP_3_/Orai1 signaling, leading to the stimulatory effects on human atrial fibroblast activity. However, the mechanism underlying the stimulatory effect of FGF-23 on PLC activation is not clear. This study did not investigate Orai1 expression after FGFR1 modulation. Thus, the regulatory mechanisms between FGF receptor 1 and PLC/Orai1 signaling remain to be elucidated.

TRPC channels are sarcolemmal cation channels and contain seven members (TRPC1-7). TRPC3 and TRPC6 have been demonstrated to regulate Ca^2+^ entry in cardiac fibroblasts and mediate cardiac fibrogenesis [21]. Our study revealed that FGF-23 did not affect the expression levels of TRPC3 and TRPC6 in cardiac fibroblasts. By contrast, FGF-23-treated cardiac fibroblasts had considerably higher TRPC1 expression levels than did control cells. Previous studies have demonstrated that TRPC1 might be activated by IP_3_ via conformational coupling with IP_3_ receptor, or following IP_3_-mediated depletion of ER Ca^2+^ stores [28,33]. Upregulation of TRPC1 contributed to the development of cardiac hypertrophy [34,35]. TRPC1 might regulate Ca^2+^ leakage from the ER in neonatal rat ventricular myocytes [36]. However, it remains unclear whether TRPC1 plays a role in modulating Ca^2+^ signaling in cardiac fibroblasts.

FGF-23 has been shown to promote myocardial fibrosis in mice with left coronary artery ligation-induced myocardial infarction, but not in sham-operated mice [13]. FGF-23 increased α-SMA expression levels in rat fibroblasts derived from obstructed kidneys. Conversely, FGF-23 had no apparent effect on α-SMA in fibroblasts isolated from normal rat kidneys [37]. A previous study also reported that FGF-23 (both 10 and 50 ng/mL) did not change the expression of α-SMA in rat ventricular fibroblasts. However, FGF-23 had a synergic effect on α-SMA upregulation in the presence of transforming growth factor β1 [38]. These findings imply the requirement for an initial injury to prime the fibroblast, thus enhancing its responsiveness to the profibrotic effects of FGF-23. Our study revealed that control and FGF-23-treated cardiac fibroblasts exhibited similar α-SMA expressions by western blot and immunofluorescence staining (Supplementary Figure S3). These findings suggest that FGF-23 may not further active myofibroblast differentiation in our experimental setting. We also found that FGF-23 did not increase collagen type IA1 and collagen type Ⅲ production in atrial fibroblasts. Thus, more markers of cardiac fibrosis are mandatory to provide laboratory evidence supporting the fibrogenic effects of FGF-23. Moreover, the tensile strength of the growth surface has a large impact on fibroblast activation and function. The atrial fibroblasts in our experiments were cultured on hard plastic dishes, which are very different from environment of the heart and have been shown to lead to fibroblast activation, inducing a potent myofibroblast differentiation and a significant expression of α-SMA in control fibroblasts [23,39,40,41]. Thus, the impact of FGF-23 on the fibroblasts cultured on a more physiologically relevant substrate remains to be elucidated.

## 4. Materials and Methods

### 4.1. Cell Culture

Human atrial fibroblasts were purchased from Lonza Research Laboratory (Walkersville, MD, USA). As described previously [22], cells were seeded on uncoated culture discs as monolayers and cultured in Dulbecco’s modified Eagle medium (Thermo Fisher Scientific, Loughborough, UK) containing 10% fetal bovine serum (Hyclone, Logan, UT, USA) and 100 U/mL penicillin–streptomycin (Thermo Fisher Scientific, Loughborough, Gibco, UK) in a humidified atmosphere of 5% CO_2_ at 37 °C. Cells used in this study were from passages 4–6 to avoid possible variations in cellular function. Cardiac fibroblasts treated without and with recombinant mouse FGF-23 (1, 5 or 25 ng/mL; R&D Systems, Abingdon, UK) in the presence and absence of an FGF receptor 1 antagonist (PD166866, 1 μM; Sigma, St. Louis, MO, USA), a free Ca^2+^ chelator (EGTA, 1 mM; Sigma), or a PLC inhibitor (U73122, 1 μM; Abcam, Cambridge, UK) for 48 h were harvested for further analysis.

### 4.2. MTS Proliferation Assay

The proliferation of cardiac fibroblasts was determined using a commercial MTS kit (Promega, Madison, WI), as described previously [23]. Cardiac fibroblasts were seeded in a 96-well culture dish at a density of 3000 cells/well. After achieving 50% confluence, fibroblasts were incubated in a medium containing FGF-23 (1, 5 or 25 ng/mL) without and with PD166866 (1 μM), EGTA (1 mM), or U73122 (1 μM) for 48 h. Cell growth was analyzed using an MTS reagent, which was added 4 h before performing the spectrophotometric analysis at an OD of 490 nm.

### 4.3. EdU-Based Proliferation Assay

EdU incorporation experiment was conducted using Click-iT^®^ Plus EdU Flow Cytometry Assay Kits (Thermo Fisher Scientific, Waltham, MA, USA) according to the manufacturer’s instructions. Briefly, cardiac fibroblasts were treated with FGF-23 (1, 5 and 25 ng/mL) for 48 h and labelled with Click-iT^®^ EdU (10 μM) for 24 h prior to the end of the treatments. After washing with 1% bovine serum albumin in phosphate buffered saline, cells were fixed, permeabilized, then incubated with 0.5 mL of Click-iT^®^ Plus reaction cocktail for 30 min to detect Click-iT^®^ EdU. Cardiac fibroblasts were resuspended in 1× Click-iT^®^ saponin-based permeabilization and wash reagent and analyzed by flow cytometry.

### 4.4. Fibroblast Migration Analysis

The migratory ability of FGF-23-treated cardiac fibroblasts without and with the co-administration of PD166866 (1 μM), EGTA (1 mM), or U73122 (1 μM) was assessed using a wound-healing assay. Briefly, confluent cells were wounded using a P200 pipette tip 8 h before the endpoint of treatments in 6-well culture plates [42]. Each gap area was analyzed using ImageJ software (National Institutes of Health). The net migration area at 48 h was subtracted from that at the baseline. Data were expressed as a fold change relative to control.

### 4.5. Western Blot Analysis

Cardiac fibroblasts were homogenized and lysed in a radioimmunoprecipitation assay buffer containing 50 mM Tris at pH 7.4, 50 mM NaCl, 1% NP40, 0.5% sodium deoxycholate, 0.1% sodium dodecylsulfate (SDS), and a protease inhibitor cocktail (Sigma), as described previously [15]. Protein concentrations were determined using a Bio-Rad protein assay reagent (Bio-Rad, Hercules, CA). Proteins were subjected to SDS–polyacrylamide gel electrophoresis under reducing conditions and electrophoretically transferred onto equilibrated polyvinylidene difluoride membranes (Amersham Biosciences, Buckinghamshire, UK). Blots were probed with primary antibodies against collagen type IA1 (#sc-293182, Santa Cruz Biotechnology, Santa Cruz, CA, USA), collagen type Ⅲ (#ab6310, Abcam), α-SMA (#ab7817, Abcam), FGF receptor 1 (#9740, Cell Signaling Technology, Beverly, MA, USA), p-FGF receptor 1 (#3476, Cell Signaling), CaMKⅡ (#GTX111401, GeneTex, Irvine, CA, USA), p-CaMKⅡ (#ab32678, Abcam), Orai1 (#4281, ProSci Incorporated, Poway, CA, USA), STIM1 (#610954, BD Transduction Laboratories, San Jose, CA, USA), TRPC1 (#ACC-010, Alomone Labs, Jerusalem, Israel), TRPC3 (#ab51560, Abcam), and TRPC6 (#ACC-017, Alomone Labs). Secondary antibodies were conjugated with horseradish peroxidase (Leinco Technology, St. Louis, MO). Bound antibodies were detected using an enhanced chemiluminescence detection system (Millipore) and analyzed using AlphaEaseFC software (Alpha Innotech, San Leandro, CA). Targeted bands were normalized to those of glyceraldehyde 3-phosphate dehydrogenase (GAPDH) (#M171-7, MBL, Japan) to confirm equal protein loading.

### 4.6. Real-Time Reverse-Transcription Polymerase Chain Reaction Analysis

Total RNAs isolated from cardiac fibroblasts were reverse-transcribed using SuperScript III reverse transcriptase (Invitrogen, Carlsbad, CA, USA). FGF receptor 1–4 mRNA expressions were analyzed through quantitative PCR by using the ABI PRISM7300 system (Applied Biosystems, Foster City, CA, USA) and SYBER Green (Applied Biosystems). Relative changes in the transcript levels of target genes were estimated from the threshold cycle (Ct) value and normalized to the respective Ct value of GAPDH (∆Ct) in corresponding samples.

### 4.7. Intracellular Ca^2+^ Imaging

Ca^2+^ imaging was performed as described previously [43]. Cardiac fibroblasts treated without and with FGF-23 (25 ng/mL for 24 and 48 h) in the presence and absence of U73122 (1 μM) for 48 h on a coverslip (1 × 1 cm^2^) were loaded with fura-2-acetoxymethyl ester (5 μM; Life Technologies, Carlsbad, CA, USA) and Pluronic F-127 (20% solution in 2.5 μg/mL dimethyl sulfoxide) in a Ca^2+^-free solution containing 120 mM NaCl, 5.4 mM KCl, 1.2 mM KH_2_PO_4_, 1.2 mM MgSO_4_, 10 mM glucose, and 6 mM HEPES (pH 7.40) for 30 min at 36 °C in a humidified incubator with 5% CO_2_. Fura-2 fluorescence images were captured using a Polychrome V monochromator (Till Photonics, Munich, Germany) mounted on an upright Leica DMI 3000B microscope (Leica Microsystems, Buffalo Grove, IL, USA) with dual excitation wavelengths of 340 and 380 nm, and an emission wavelength of 510 nm. Fura-2 images were analyzed using MetaFluor software (version 7.7.9.0; Molecular Devices, Sunnyvale, CA, USA). The ratio of emitted fluorescence due to excitation at 340 nm (F340) to 380 nm (F380) was used as a marker of the relative intracellular Ca^2+^ levels. Cells were first exposed to the Ca^2+^-free solution, followed by ER Ca^2+^-ATPase inhibitor (thapsigargin, 2.5 μM; Sigma) co-treatment for ER Ca^2+^ store depletion to assess ER Ca^2+^ release. After the intracellular Ca^2+^ surge from ER Ca^2+^ leak induced by thapsigargin returned to a steady-state, the extracellular Ca^2+^ concentration was then increased to 2 mM to measure Ca^2+^ entry. Acute Ca^2+^ response to FGF-23 was assessed in the presence of extracellular Ca^2+^ (1.8 mM) without and with pretreatment of U73122 (1 μM for 5 min) or 2-APB (50 μM added 5 min prior to recording) [32,44,45]. To dissect ER Ca^2+^ release from external Ca^2+^ entry, cardiac fibroblasts were stimulated with FGF-23 (25 ng/mL) in a Ca^2+^-free condition, followed by restoration of extracellular Ca^2+^ concentration to 1.8 mM. The integrative (area under the curve) values of fluorescence intensities (F340/F380) corresponding to ER Ca^2+^ release and Ca^2+^ entry were calculated for analysis respectively.

### 4.8. IP_3_ Measurement

The cell lysates of cardiac fibroblasts with and without FGF-23 (25 ng/mL) treatment for 24 h and 48 h were collected for the IP_3_ measurement by using an ELISA kit (Bioassay Technology Laboratory, Shanghai, Yangpu, China) according to the manufacturer’s protocol. Data were normalized to protein concentrations and expressed as a fold change relative to control.

### 4.9. Statistical Analysis

All quantitative data are expressed as the mean ± standard error of the mean. Statistical significance in cardiac fibroblasts under different conditions was determined using a one-way repeated analysis of variance with a post hoc Tukey’s test and the paired or unpaired *t*-test. A *p* value of <0.05 was considered to indicate a statistically significant difference.

## 5. Conclusions

FGF-23 activates FGF receptor 1 and subsequently PLC/IP_3_ signaling pathway, leading to an upregulation of Orai1 and/or TRPC1-mediated Ca^2+^ entry and thus enhancing the proliferative and migratory abilities of human atrial fibroblasts.

## Data Availability

The data presented in this study are available on request from the corresponding author.

## Supplementary Materials

The following are available online at www.mdpi.com/article/10.3390/ijms23010166/s1.
