# Supplementary material for: Fibroblast Growth Factor 23 Stimulates Cardiac Fibroblast Activity through Phospholipase C-Mediated Calcium Signaling
Source: Int J Mol Sci. 2021 Dec 23;23(1):166. doi: 10.3390/ijms23010166 (PMC8745152; doi:10.3390/ijms23010166)
Supplement: Supplementary file 1 [file ijms-23-00166-s001.zip › ijms-1447056-supplementary.pdf]

Supplemental Figures

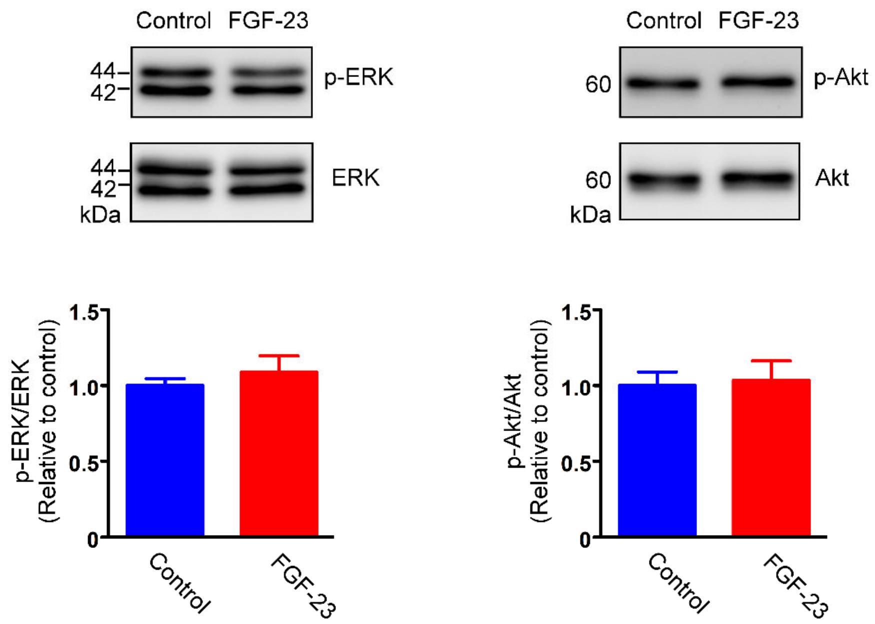

Figure S1. Fibroblast growth factor (FGF)-23 on extracellular signal-regulated kinase (ERK) and protein kinase B (Akt) signalings in human atrial fibroblasts. Representative immunoblots of total and phosphorylated-ERK (p-ERK) (left panel), total and phosphorylated-Akt (p-Akt) (right panel) in cardiac fibroblasts without and with FGF-23 (25 ng/mL) treatment for 15 minutes. FGF-23 (25 ng/mL) did not change phosphorylation levels of ERK and Akt in human atrial fibroblasts. Ratio of p-ERK to ERK or p-Akt to Akt was expressed as a fold change relative to control (n = 5). Paired *t*-test was used to compare the control and FGF-23-treated human cardiac fibroblasts. The antibodies used for western blot were ERK (#9102, Cell Signaling), p-ERK (#4370, Cell Signaling), Akt (#4685, Cell Signaling), and p-Akt (#4060, Cell Signaling).

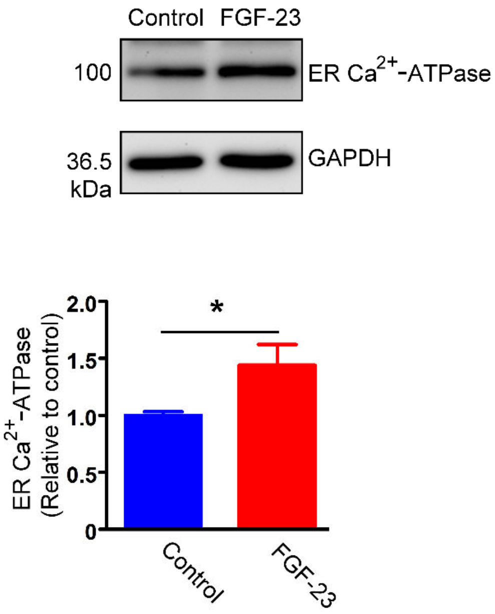

Figure S2. Fibroblast growth factor (FGF)-23 on protein expression levels of endoplasmic reticulum calcium ATPase (ER Ca^{2+}-ATPase) in human atrial fibroblasts. Representative immunoblots and average data of ER Ca^{2+}-ATPase in cardiac fibroblasts without and with FGF-23 (25 ng/mL) treatment for 48 hours. FGF-23 (25 ng/mL)-treated human atrial fibroblasts had a greater expression of ER Ca^{2+}-ATPase than did control cells. Data was expressed as a fold change relative to control (n = 5). Paired *t*-test was used to compare the control and FGF-23-treated human cardiac fibroblasts. * $p < 0.05$. The antibodies used for western blot were ER Ca^{2+}-ATPase (#sc-8095, Santa Cruz) and glyceraldehyde 3-phosphate dehydrogenase (GAPDH) (#M171-7, MBL).

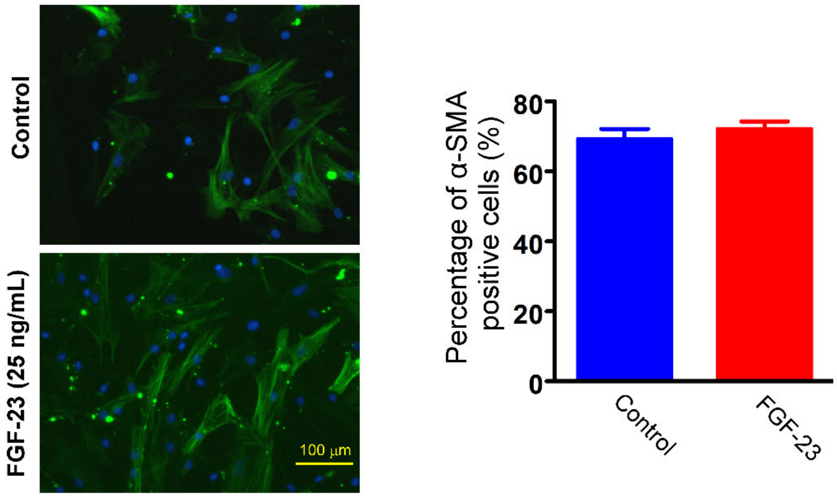

Figure S3. Fibroblast growth factor (FGF)-23 on the expression of α-smooth muscle actin (α-SMA) in cultured human atrial fibroblasts. Representative photograph of immunofluorescence microscopy imaging (left panel) and average data (right panel, n = 5) of α-SMA expression in cardiac fibroblasts without and with FGF-23 (25 ng/mL) treatment for 48 hours. The expression of α-SMA was assessed by immunofluorescence staining (green) as described previously (Chen et al., 2021, Int J Mol Sci, doi:10.3390/ijms22020842). Nuclei were counterstained by DAPI (blue). The percentage of α-SMA positive cells were similar between control and FGF-23 (25 ng/mL)-treated human atrial fibroblasts. Data were expressed as a ratio of the number of α-SMA positive cells to the number of DAPI positive cells. Paired *t*-test was used to compare the control and FGF-23-treated human cardiac fibroblasts. Scale bar represents 100 μm.
